# The Five-Year Outcomes of Breast Cancer Surgical Management at the Colentina Surgical Clinic, Bucharest, Romania: A Descriptive Retrospective Analysis Between 2019 and 2023

**DOI:** 10.3390/life15010092

**Published:** 2025-01-14

**Authors:** Cristian Botezatu, Daniel-Ovidiu Costea, Martina Nichilò, Angela Madalina Lazar, Dan Andraș, Mircea-Ion Radu, Bogdan Mastalier

**Affiliations:** 1 General Surgery Department, Carol Davila University of Medicine and Pharmacy, 8 Eroii Sanitari Blvd., 050474 Bucharest, Romania; cristi_botezatu2001@yahoo.com (C.B.); angelalazar.2008@yahoo.com (A.M.L.); dan.andras@drd.umfcd.ro (D.A.); bogdanmastalier@yahoo.com (B.M.); 2 General Surgery Clinic, Colentina Clinical Hospital, 020125 Bucharest, Romania; raduionmircea@gmail.com; 3 2nd General Surgery Clinic, County Clinical Emergency Hospital, General Surgery Department, Ovidius University, 145 Tomis Boulevard, 900591 Constanța, Romania; danielcostea@gmail.com

**Keywords:** breast cancer, surgical treatment, mastectomy, early diagnosis

## Abstract

**Background:** Breast cancer still represents the most commonly diagnosed cancer among women, accounting for 12.5% of all new annual cancer cases worldwide. In Romania in 2020, breast cancer was the most common, with a share of new cases of 26.9%, far behind the rates of colon cancer (11.8%) and cervix cancer (7.5%). The aim of this study is to reveal five years of experience in treating breast cancer at the Surgical Clinic of the Colentina Hospital in Bucharest, Romania. **Methods:** Retrospective analysis, including 68 patients admitted to our clinic between January 2019 and December 2023 undergoing modified radical mastectomy, sectorectomy, or subcutaneous mastectomy. **Results**: Madden-modified radical mastectomy with total excision of the axillary lymph nodes accounted for 77.94% of surgeries, with a complication rate of 13.2%, represented by lymphedema of the ipsilateral arm and prepectoral seroma. **Conclusions:** Continuous innovation regarding early diagnosis and treatment methods in our surgical clinic will, hopefully, contribute to improving the outcomes of our patients.

## 1. Introduction

Breast cancer is the most common type of cancer worldwide in women, accounting for 11.6% of total new cases, followed by colorectal cancer, prostate cancer, and stomach cancer [1,2]. Furthermore, while the incidence of all types of cancer worldwide has been steadily decreasing since 1991 due to factors such as an increase in cancer awareness and a reduction in favoring factors [3], breast cancer incidence has shown a slow but statistically significant increase in the last decade for every age group (the highest being registered in women younger than 50 years) [2,4] and every world region [4]. Rates are higher in countries with a higher HDI (human development index, a composite measure of life expectancy, education, and wealth and a more useful comparator between countries than income alone) [5] in comparison to countries with a lower HDI [4,6,7] and also show an unequal distribution among White and Black women (the former showing an increase in incidence compared to the latter) [8]. Despite this increase in breast cancer incidence being amenable to earlier detection and the creation of screening programs in several countries [9,10], these data are still very alarming, especially since breast cancer mortality has been decreasing at a much slower pace in the last decade [2,3,8] and is higher in low HDI countries [1,4,6,9] and Black women compared to White women [8].

Consistently with the data registered for female individuals, male breast cancer has registered in the last decade an increase in incidence from 7% to 10%, even though male breast cancer remains a rather uncommon disorder (only 1% of all breast cancers worldwide affect the male population) [11,12]. Also, in accordance with their female counterparts, breast cancer in men has shown a decrease in its associated mortality, although registering much higher mortality compared to women (60% excess mortality) [11,12]. Predictors for mortality in this specific category are several, including the cancer stage at the moment of diagnosis, lower breast cancer awareness among the male population as opposed to the female one [12], and important biological differences between male and female breast cancer (such as the increased prevalence of BRCA2 germline mutations or the prevalence of hormone receptor-positive cancer), which imposes the need for additional research on male breast cancer and a tailored therapeutical approach for this specific category [13].

Reducing the mortality associated with breast cancer has become of key importance worldwide and is one of the main challenges to treatment. In the latest update to its manual on cancer staging, the American Joint Committee on Cancer introduced a new staging model for breast cancer: prognostic staging. This new model, to be used only in those practices in which biomarker assessment is routinely performed for breast cancer, combines the classic anatomic TNM staging with tumor grade and the status of three important prognostic factors, namely human epidermal growth factor receptor 2 (HER2) and two hormonal receptors (ER (estrogen receptor) and PR (progesterone receptor)) [14].

This change is a significant one considering that the management of breast cancer is no longer selected only according to its anatomical stage but also takes into account its specific molecular and biological characteristics. This is mostly due to the groundbreaking discoveries in the field of oncological treatments, which now allow more specific targeted therapy with smaller effects on non-cancerous cells while preserving the same efficacy or even better compared to traditional therapies [15]. At the same time, surgery for breast cancer has evolved as well in recent decades by developing techniques that are less invasive and more accurate, thus presenting several procedures that are collectively known as breast-conserving surgery (BCS) as opposed to classic total mastectomy, which has also undergone a process of evolution since the development of the Halsted procedure [15].

The existence of such a large array of therapeutical options for patients with breast cancer has resulted in a shift from standardized therapeutical regimens to a more precise approach that takes into account the specificity of the patient, the tumor, and the available treatment, thus developing the idea of a tailored treatment plan [16].

According to the NCCN’s (National Comprehensive Cancer Network) latest Clinical Practice Guidelines in Oncology, patients with an operable early-stage invasive breast cancer, defined as stages IA, IIA, and IIB [14], benefit from undergoing surgical treatment first with or without adjuvant therapy and considering neoadjuvant treatment only in a small number of cases, such as patients with more aggressive diseases (HER2-positive or triple-negative tumors equal or greater than 2 cm in size or with clinically active axillary lymph nodes) [17,18].

Neoadjuvant systemic therapy has some clear and known benefits, such as the following:

- Facilitates breast conservation;
- Can render inoperable tumors operable;
- Treatment response provides important prognostic information at an individual patient level, particularly in patients with TNBC or HER2-positive breast cancer;
- Allows time for genetic testing;
- Allows time to plan breast reconstruction in patients electing mastectomy;
- Allows time for delayed decision-making for definitive surgery.

Moreover, it may allow sentinel lymph node biopsy alone if initial cN+ becomes cN0 after preoperative therapy, may allow for more limited radiation fields in patients with cN+ who become cN0/pN0 after preoperative therapy, and provides an excellent research platform to test novel therapies and predictive biomarkers [17].

However, not all patients can undergo neoadjuvant therapy and thus, must be carefully selected [19]. According to the NCCN guidelines [17], candidates for Preoperative Systemic Therapy are:

- Patients with inoperable breast cancer;
- Selects patients with operable breast cancer with HER2-positive disease or TNBC, if ≥cT2 or ≥cN1 or with large primary tumor relative to breast size who desires breast conservation or in patients with cN+ disease likely to become cN0 with Preoperative Systemic Therapy.

Non-candidates for Preoperative Systemic Therapy are:

- Patients with extensive in situ disease when the extent of invasive carcinoma is not well-defined;
- Patients with a poorly delineated tumor extent;
- Patients with tumors that are not palpable or clinically assessable.

While surgery remains the first line of treatment in early-stage breast cancer [17,18], its techniques and procedures have drastically changed and transformed since the historic radical mastectomy described by Halsted in the 1890s, which involved the excision of the entire breast, pectoralis muscles and lymphadenectomy [18,20].

Options of surgical treatment currently include:

- Surgical treatment of the primary breast tumor: BCS or mastectomy (radical or simple);
- Management of the axilla: sentinel lymph node biopsy or axillary lymph node dissection.

### 1.1. BCS (Breast-Conserving Surgery)

There are various terms used to describe breast-conserving surgery, including quadrantectomy, lumpectomy, or partial mastectomy. Quadrantectomy involves excision of the tumor, including a 2- to 3-cm margin, pectoralis fascia, as well as the overlying skin. On the other hand, lumpectomy indicates a less generous tissue excision with a 1-cm margin [20,21]. BCS thus refers to all the procedures that allow patients to preserve the breast still respecting the oncological safety offered by mastectomy [17,21] only if it is followed by radiotherapy, which has been demonstrated to have a protective effect against local recurrence and death with women not undergoing radiotherapy after BCS being more than twice as likely to die or to experience local recurrence [22]. Indications for breast-conserving surgery include DCIS/Tis and T1-2 tumors, if no contraindications to adjuvant radiation therapy, small tumors amenable to resection with clear margins, and acceptable cosmesis. Various studies have offered various tumor sizes as cutoff values for the eligibility of the patient to undergo BCS. However, treatment should be patient-specific, and women with larger breasts may still be eligible for breast-conserving surgery with a large tumor if the patient has sufficient breast size [20], preferring mastectomy followed by breast reconstruction if the breast-conserving treatment cannot ensure oncologic safety or a good aesthetic result [23]. BCS is contraindicated to female patients who are pregnant and would require radiotherapy during pregnancy that present with diffuse suspicious or malignantly appearing microcalcifications on mammography, a widespread disease that cannot be covered by local excision of a single region or segment of the breast tissue with a satisfactory cosmetic result, and have diffusely positive pathological margins [17,20]. Overall, BCS remains one of the best surgical treatments, with many advantages when compared to total mastectomy in patients with early-stage breast cancer, such as lower complication rates, shorter hospital stays, reduced need for pain medication, and faster return to work and social life. Moreover, it excludes the need for breast reconstruction with its associated complications, higher chance of additional revisional interventions as well as poorer cosmetic outcome in the case of adjuvant irradiation of the breast [24].

### 1.2. Mastectomy

While the historic radical mastectomy described by Halsted is no longer employed in modern medicine, mastectomy, in all its modified forms and types, is still largely used in the treatment of breast cancer, including the modified radical mastectomy described by Madden (which spares the pectoralis muscles while still including the excision of the level I-III axillary lymph nodes) as well as the simple mastectomy which implies the removal of the whole breast tissue without necessitating a complete axillary node dissection [20]. Multiple indications for mastectomy exist. These indications refer to patients with advanced disease, including T2 (greater than 5 cm) tumors, multicentric or multifocal disease, chest wall involvement, or inflammatory breast cancer, which is generally considered T4 disease. Another indication for mastectomy includes patients with Paget disease, indicating tumor cells of the epidermis of the nipple-areolar complex. Mastectomy is contraindicated in patients with known metastatic disease, individuals with a poor performance status who are unable to tolerate general anesthesia, as well as in certain patients with advanced locoregional disease who require neoadjuvant treatment to downstage tumor before any surgical resection [20].

Classic mastectomies generally involve an elliptical excision of the breast, which includes the nipple-areolar complex. However, two additional forms of mastectomy exist, skin-sparing (SSM) and nipple-sparing (NSM), which usually imply an immediate reconstruction of the breast [18,20]. Of these two techniques, the latter is most frequently performed because of a slightly superior outcome from the aesthetic as well as psychological point of view [25]. In SSM the surgeon removes the gland but leaves most of the breast skin to create a pocket that is filled with a breast implant or the patient’s tissue. NSM is closely similar to SSM, but is the real conservative innovation in that the nipple-areola complex is preserved as well as the skin [25]. The main concerns about the NSM are surgical and oncologic. The main surgical concern is nipple necrosis, which can lead to a poor cosmetic outcome and even loss of the nipple-areola complex. On the other hand, the oncologic concern is the possibility of leaving behind glandular and ductal tissue, which could be a source of cancer recurrence [26]. Such a risk can be reduced by sending a section of the tissue from the base of the nipple for evaluation of the clear surgical margin during surgery, and if the nipple is affected at the surgical margin, the entire nipple-areola complex should be removed thus turning the NSM in an SSM [25,26]. Both surgical and oncological concerns are significantly reduced by close collaboration between an experienced breast surgeon and a plastic surgeon, as well as by carefully selecting suitable patients to undergo this type of procedure [25,27]. The NCCN 2024 guidelines [17] suggest that NSM is oncologically safe as long as the following indications are met: early-stage breast cancer, DCIS, risk-reducing procedures (prophylactic mastectomy in women with a BRCA mutation or strong family history of cancer) [28], and in some locally advanced invasive cancers (i.e., with complete clinical response to preoperative chemotherapy and no nipple cancer involvement). Moreover, the ideal candidate for NSM is a patient with small breasts, BMI < 30 kg/m^2^, no or minimal ptosis, and who is not an active smoker [26,27]. Contraindications for NSM include preoperative clinical or radiographic evidence of nipple involvement, including Paget disease, bloody nipple discharge associated with malignancy, IBC, and/or imaging findings suggesting malignant involvement of the nipple or subareolar tissues. Surgical techniques and incisions are multiple, depending on the size of the breast, the location of the tumor, etc.

Overall, women undergoing NSM experienced significantly less postoperative psychological distress related to body image compared to women who underwent a non-NSM, felt less mutilated, and had greater sexual well-being [26].

### 1.3. Management of the Axilla

In breast cancer therapy, it is imperative to consider the axillary lymph nodes. Since ancient times, it has been demonstrated that breast cancer poses a risk of metastatic dissemination and extension to the axilla. Even though Halsted was not the first surgeon to introduce the concept of axillary dissection as part of the surgical treatment of breast cancer, he was the one who standardized it when he proposed radical mastectomy, thus achieving the removal of the breast, along with the pectoral muscles and axillary lymph nodes en bloc [15,29]. While ALND (axillary lymph node dissection) remains the current standard procedure and best option in patients with clinically positive axillary lymph nodes, the same cannot be said for women with clinically negative axilla. Thus, as with mastectomy, axillary management has evolved and gone through a process of revision, leading to the introduction of the concept of sentinel lymph node (SNL) and sentinel lymph node biopsy (SNB) [19,30]. The need to determine whether ALND is mandatory in all breast cancer patients, to perform ALND only in those patients who need it, and to evaluate the long-term oncological safety of a less invasive procedure is a major concern in the medical community due to its association with postoperative complications such as lymphedema, paresthesia, and limited mobility of the arm and hand [31]. One of the first studies to find that SNB has the same safety and effectiveness in breast cancer axillary node staging and prognosis as ALND was NSABP B-32 [32]. After that, ACOSOG Z0011 demonstrated that in patients with cT1-T2, clinically negative axilla (cN0), undergoing BCS or mastectomy with subsequent whole breast irradiation, which have less than 3 positive SNLs on biopsy, we can avoid performing ALND because there is no difference in overall survival as well as in axillary recurrence at 10 years, between those with and without ALND [33]. Presently, the consensus is that SNL is the best choice in patients with early breast cancer and clinically negative axilla and that the routine use of ALND is no longer justified [29,33,34]. Further scientific research on SNB attempts to implement selection criteria for patients not needing to undergo ALND and newer and better techniques for identifying axillary SNLs [35,36].

## 2. Materials and Methods

We conducted a retrospective descriptive study on a sample of 68 breast cancer patients admitted to the Surgery Clinic of Colentina Hospital, Bucharest, Romania, between January 2019 and December 2023. The small number of patients is determined by the fact that our hospital was designated as a COVID-only hospital during the pandemic.

The study was conducted in accordance with the Declaration of Helsinki, the protocol being approved by the Institutional Review Board (or Ethics Committee) of the Colentina Clinical Hospital, Number 12/17 August 2024.

We analyzed the distribution of patients by gender, age group, and area of origin (urban/rural). Next, we showed the distribution of patients according to the data obtained during the clinical examination: tumor location and size, presence of local pain, adhesion to deep tissue, skin and nipple retraction, ulcerations, nipple discharge, and the existence of axillary lymphadenopathy. We also included data on the distribution of patients according to the data obtained during the paraclinical examination: the TNM classification, histopathological analysis, immunohistochemical analysis (estrogen receptor-ER, progesterone receptor-PR, HER-2 receptor), clinical prognostic staging, and the CA 15-3 levels.

Finally, we analyzed data regarding the treatment performed, both surgical and systemic, postoperative complications and evolution.

The aim of our study was to present the experience of our clinic in the surgical treatment of breast cancer, the results obtained through various approaches, as well as the difficulties and controversies in dealing with such a pathology.

## 3. Results

The analysis of the study group began with the distribution of patients by sex and age groups.

**Figure 1:**
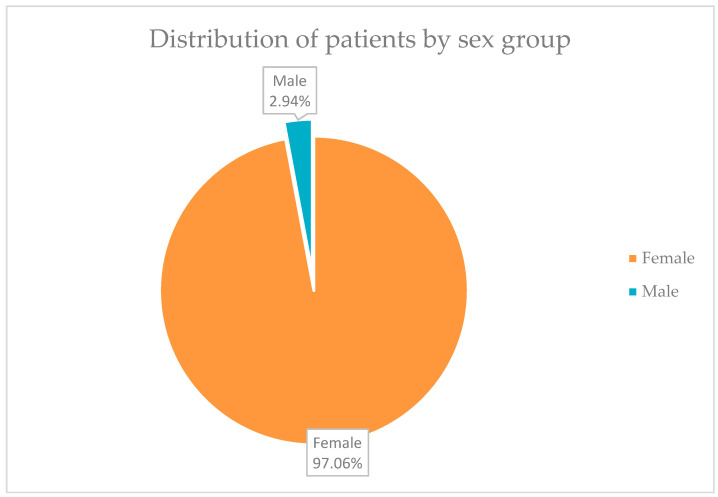
Distribution of patients by sex group.

In Figure 1, it can be seen that 66 of the 68 patients, in total, a percentage of 97.06%, were represented by women, and only 2 patients, representing 2.94% of the group, were men.

**Figure 2:**
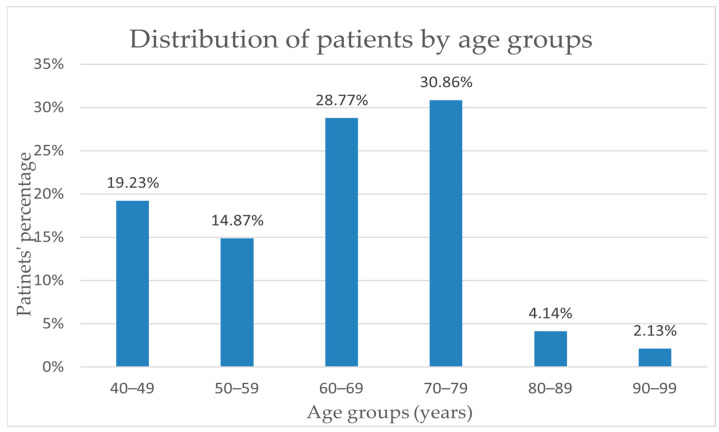
Distribution of patients by age groups.

For the study group of 68 people, the average age was 64.16 years old, and the median was 67 years old. An important increase in incidence can be observed starting at the age of 60, as 30.86% of diagnosed cases are in the age range of 70–79 years and a percentage of 29% in the age range of 60–69 years old. The incidence rate in the 40–49 age group (19.23%) is higher than the rate of 14.87% in the 50–59 age group. Also, an obvious decrease in breast cancer incidence can be noted only after the age of 80, where the incidence reaches almost 6% of the total number of patients included (Figure 2).

**Figure 3:**
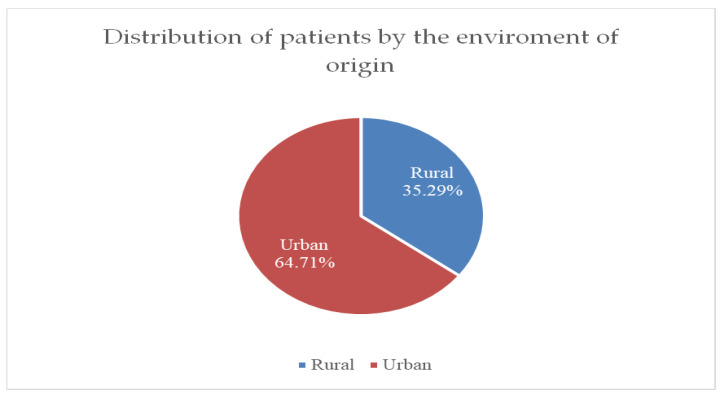
Distribution of patients by environment of origin.

Figure 3 shows that the incidence of breast cancer cases is higher in urban areas, at 64.71%, compared to rural areas, at only 35.29%.

**Figure 4:**
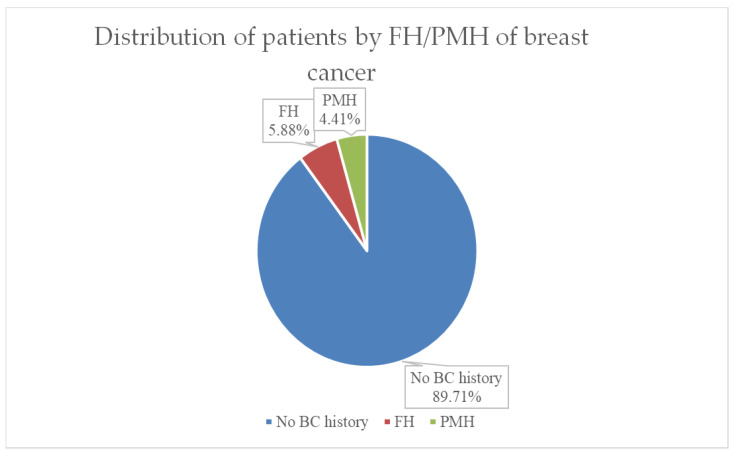
Distribution of the patients by FH/PMH of breast cancer.

Both family medical history (FMH) and personal history of breast cancer (PMH or PMR) have important value in assessing prognosis and therapy in breast cancer. As can be seen in Figure 4, in our group of patients, 90% had no history of breast cancer, 6% (representing 4 cases) had first-degree relatives of breast cancer, and 3 cases (representing 4%) of the group had a recurrence after breast cancer in the past.

In the group of 68 patients who presented to the clinic, 95.5% (65 patients) were diagnosed with breast tumors for the first time, these patients having no personal pathological history in the field of breast cancer. The remaining 4.5% presented for breast cancer recurrence, all tumors being located in the contralateral breast.

Menopause is an important factor in assessing breast cancer risk, prognosis, and treatment. In our group of 68 patients, 66 are women. In 51 of them, representing a total of 77.27%, menopause was already established at the time of diagnosis. In the remaining 22.73%, women were diagnosed with breast cancer during the premenopausal period.

In our patient group, the average age of women diagnosed with breast cancer in whom menopause has already set in is 64.5 years, and the median is 52.5 years. For the 15 patients diagnosed with breast cancer while still in pre-menopause, the average age is 45.4 years, and the median is 41.5 years.

Regarding tumor location, right versus left side, 52.82% of patients had the primary tumor located in the left breast and the remaining 47.18% in the right breast.

Table 1 shows the distribution of tumor location in the 5 quadrants of the breast. In the study group of 68 patients, the most frequently affected is the upper-outer quadrant, in 30.88% of cases. Next in frequency, with a percentage of 16.17%, are the lower-outer and upper-inner quadrants. Lower-inner and central quadrants are least frequently affected in only 13.23% of cases. The localization of multiple tumor formations in at least two quadrants, thus multi-centricity, was encountered in 10.29% of cases.

The primary tumors were of various sizes. The mean of the values was 2 cm, and the median was 2 cm. As can be seen in Table 2, most of the tumor formations identified in our group of patients, in 47.05% of cases, had a size of approximately 2 cm. The next most frequent size was about 3 cm, found in about 23.52% of cases.

Pain is an important symptom in the diagnosis of breast cancer, but it appears quite late. The incidence of breast pain in our group of 68 patients was present in 40 patients (58.82%).

Another important clinical sign in the diagnosis of breast cancer is the presence of skin or nipple retraction. During the clinical examination, both skin and nipple retraction can be observed, especially in the case of tumors located more superficially, towards the skin or the areola, and especially in the advanced stages of the disease. Skin and/or nipple retraction was found in 30 patients in the study group (44.11%).

Regarding tumor adhesion to deep tissue, this clinical sign was present in 82.35% of patients in our study group (56 patients).

The presence of skin or nipple ulcerations is a sign of advanced disease and severity. In our group of 68 patients, 4 of the patients (5.88%) presented with ulcerations, of which 2 patients presented with nipple ulcerations and the other 2 presented with skin ulcerations.

Also, 4 patients, representing 5.88% of the total, presented with nipple discharge. Of these, only one patient presented with serous-hemorrhagic nipple discharge. In our cohort, all patients who were identified with nipple discharge were diagnosed with G3 invasive breast carcinoma.

The clinical examination performed on the patients identified palpable axillary lymph nodes in 58 of the 68 patients (85.29%).

All patients received preoperative mammography, ultrasound, and CT scan. Based on ultrasound examination, 29 of the patients were detected as BIRADS 4, and 31 as BIRADS 5. Only 8 patients were identified as BIRADS 3, and core needle biopsy (CNB) was performed in these patients.

The CA 15-3 marker was found to be elevated in only 14.23% of the patients, once again emphasizing its important role in the follow-up of patients with breast cancer rather than in diagnosing the disease.

**Figure 5:**
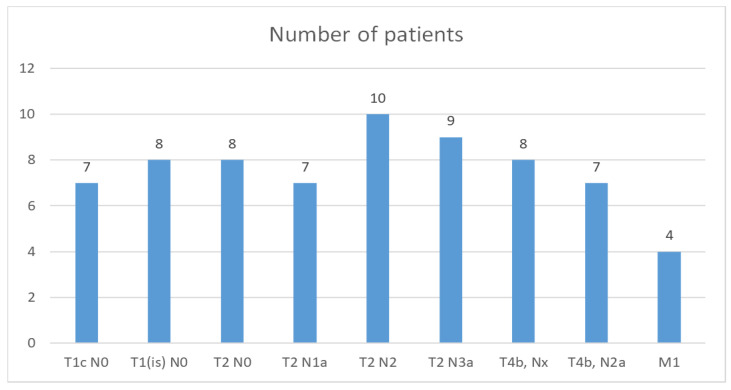
Distribution of patients according to TNM Staging.

The TNM classification found in our study group is shown in Figure 5.

**Figure 6:**
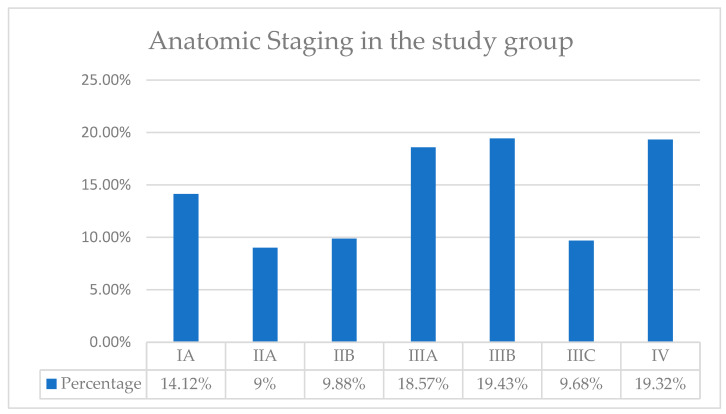
Distribution of patients according to Anatomic Staging.

Based on the TNM classification, we then determined the anatomical stages of our patients, as seen in Figure 6. Thus, in our patient group, the highest incidence of patients was in Stages IIIA, IIIB, and IV, with a percentage of approximately 19%. We identified the lowest incidence for stage IIA, with a percentage of only 9%.

**Figure 7:**
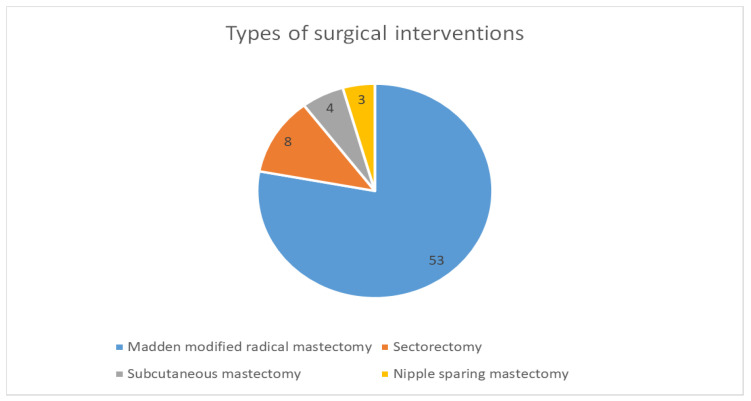
Distribution of patients according to the types of surgical interventions performed.

Regarding the types of surgical interventions performed for our group of 68 patients, a total of 53 patients (77.94%) benefited from modified Madden-type radical mastectomy with radical axillary lymph node dissection. Sectorectomy was performed in 8 cases, subcutaneous mastectomy in 4 patients, and nipple-sparing mastectomy in 3 patients, as seen in Figure 7.

Postoperative evolution was favorable in 59 of the patients (86.7%). As postoperative complications, lymphedema of the ipsilateral arm was encountered in 6 patients and prepectoral seroma in 3 patients. It should be noted that all complications that occurred followed modified radical mastectomy with lymph node excision. No complications were reported following the other types of surgical interventions.

Following histological analysis, the histological grade of the tumor was established. Of our group of 68 patients, only one was diagnosed with well-differentiated tumor grade G1, with a favorable prognosis. The majority of the patients, representing 54.11%, were classified in Grade G2, i.e., Intermediate Combined Histologic Grade, moderately differentiated, with a moderately favorable prognosis. A percentage of 44.41% of patients were classified in Grade G3, i.e., High Histological Grade, poorly differentiated with unfavorable prognosis.

We investigated the presence of the HER-2, ER, and PR. The incidence of HER-2 receptor positivity was 67.23%, and for 32.77% of cases, HER-2 was negative. The estrogen receptor was positive in most of the cases, 75.48%, and negative in 24.52% of cases. Progesterone receptors were positive in 42.84% of cases, while most of the cases had negative progesterone receptors in 57.16%.

**Figure 8:**
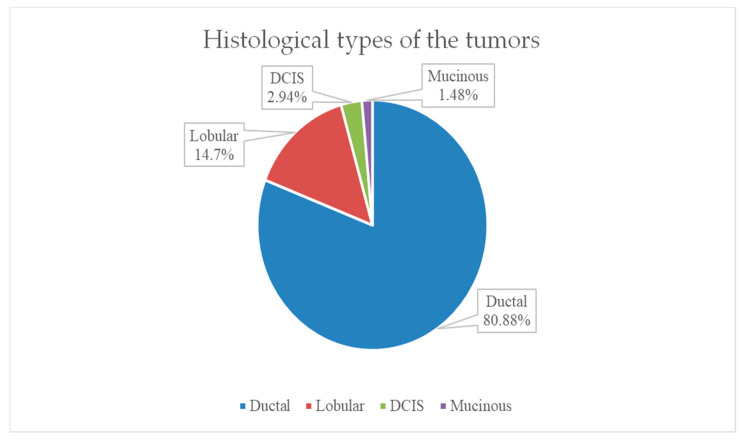
Distribution of patients by histological type of tumor.

The most frequent histological tumor type encountered in the study group was invasive ductal carcinoma in 80.88% (55 patients). The second most frequent histological type was invasive lobular carcinoma in 10 patients, representing 14.7% of our group. Only one case of special invasive carcinoma–mucinous type was identified, and 2 cases of ductal carcinoma in situ (DCIS) were also identified, as shown in Figure 8.

Regarding oncological treatment, all patients underwent postoperative chemotherapy, and 9 patients underwent neoadjuvant chemotherapy. The chemotherapy regimen in neo-adjuvant therapy was represented by 8 cures of Cisplatin.

## 4. Discussion

Female patients in the study group represented the percentage majority. This is according to data in the specialized literature, which tells us that female gender is a major risk factor for breast cancer. Breast cancer is still the most common type of cancer among women, with 1 in 8 women being diagnosed with the disease during their lifetime. Precisely for this reason, public health policies have made great efforts for the early diagnosis of breast cancer by introducing, even decades ago, breast cancer screening at a broad level in the population [37].

According to data from the literature, we also identified in our group of patients that the incidence rate is higher in women over 50 years old compared to those under 50 years old. For our study group, the incidence rate in the 40–49 age range of 19.23% is higher than the rate of 14.87% in the 50–59 age range. According to data in the specialized literature, the incidence of breast cancer increases with age, doubling every 10 years until menopause, where a decrease can be observed, the average age of onset of menopause being 51 years [37].

The incidence of breast cancer cases was higher in the urban areas compared to rural areas, a fact that is also noted in the specialized literature. Studies estimate that the incidence of breast cancer in urban areas is almost 76%, while in rural areas, it is only 24%. This may be due to several factors. The addressability of patients from rural areas and their access to medical services is lower compared to patients from urban patients. Socio-economic factors may contribute to a differentiation in patient prognosis. Patients who come from rural, poor areas will be less able to afford the costs of treatments, which leads to late presentations to the doctor when the disease is already in more advanced stages. In addition, patients in rural areas often do not have medical insurance to pay for multiple medical investigations, which makes early diagnosis much more difficult. Moreover, patients in rural areas give up less of their daily activities and work to go to medical appointments. Additionally, awareness and knowledge about breast cancer are much higher in urban areas than in rural areas. Thus, urban patients are more likely to be aware of the signs and symptoms of breast cancer. It was thus found that improving awareness and education in rural areas will also lead to better outcomes for these patients. On the other hand, urban lifestyle is often Western, sedentary, with a hypercaloric diet and high alcohol consumption, which is frequently correlated with the development of other risk factors: obesity, often abdominal, and chronic hyperinsulinism. In contrast, the lifestyle of people in rural areas is more compatible with a Mediterranean type, with a diet rich in fruits, vegetables, and cereals and low in saturated fat and carbohydrates, which is considered to be a protective factor. It is known from the literature that socio-economic factors, along with clinical and genomic factors, play a very important role in the disproportionality of the disease prognosis between patients from rural and urban [38,39].

A family history of breast cancer is an important risk factor for this type of cancer. The risk is almost 4 times higher if the mother or sister is affected and is up to 5 times higher in women who have two or more first-degree relatives diagnosed with breast cancer, especially at young ages, under 50 years. In the study group, 4 patients had a family history of breast cancer in first-degree relatives. Two of the patients were 41 and 43 years old, respectively, so under 50 years old, thus with a significantly higher risk of developing a form of breast cancer, as the studies also say.

Menopause is an important factor in breast cancer risk assessment, prognosis, and treatment. In our study group of 68 patients, 66 are women. In 51 of these, menopause was already established at the time of diagnosis. In the remaining female patients, menopause was not established, the women being in the premenopausal period. In our patient group, the average age for women diagnosed with breast cancer in whom menopause has already set in is 64.5 years, and the median is 52.5 years. For the 15 patients diagnosed with breast cancer while still premenopausal, the average age is 45.4 years, and the median is 41.5 years. Numerous studies indicate that late menopause is an important risk factor for breast cancer. Postmenopausal women have a lower risk of breast cancer compared to premenopausal women. The risk increases by 3% each year after menopause, whether natural or surgically induced. Thus, women who reach menopause at age 55 have a 30% higher risk of developing breast cancer compared to those who enter menopause at age 45 [40].

Regarding the location of the tumor on the right or left breast, in our group of 68 patients, 52.82% had the primary tumor located in the left breast and the remaining 47.18% in the right breast. It is known from the literature that the left breast is 5–10% more likely than the right to develop breast cancer. Thus, the data we collected are in accordance with those in the specialized literature.

As far as the location of the primary tumor in one of the quadrants of the breast, we showed that in our group, the most frequently affected was the upper-outer quadrant. Next in frequency were the lower-outer and upper-inner quadrants. The lower-inner and central quadrants were the least frequently affected. The location of multiple tumor formations in at least two quadrants, thus multicentricity, was encountered in 10.29% of cases. In many studies, it is demonstrated that the most frequent place of occurrence of breast cancer is in the upper-outer quadrant, and the least frequent is in the lower-inner quadrant [41,42].

The majority of primary tumors identified in the patient group were approximately 2 cm in diameter. The next most frequent diameter was about 3 cm. Numerous studies consider that the 2 cm size of the primary tumor occupies a special niche in oncology, being one of the most frequently diagnosed sizes [43]. The size of 2 cm is at the border between stage I and II for cancers without lymph node involvement, and the border between stage II and III for cancers with positive lymph node involvement. The size of the primary tumor and the status of the nodules are two of the most important parameters in determining the prognosis and planning the treatment of patients with breast cancer.

We analyzed the presence of breast pain at the time of diagnosis. The incidence of breast pain in our group of 68 patients is 58.82%. In the literature, pain is reported in approximately 30% of cases at presentation, while post-treatment pain occupies a much more important place, being found in approximately 60% of patients, especially in the case of those operated on and irradiated. The higher percentage in our study may also be due to the average age at presentation of 64 years, which may also imply a later presentation at a first checkup when the disease is already more advanced and there is a greater probability for the pain to occur. In addition, 3 patients from our group presented for recurrence, and 2 patients are 41 and 43 years old. In all these patients, the disease is more likely to be more aggressive [44].

Skin and/or nipple retractions were encountered at presentation in 44.11% of our patients. Therefore, the majority of patients did not present this sign. Also, in the literature, the incidence of this sign is about 36%. Thus, we can say that the data obtained by us are comparable to those in the literature.

We determined that most of the patients in the study group had palpable adhesions to the deep tissue. This high percentage may also be determined by patients’ later presentation to the doctor and the type of cancer in more advanced stages. However, palpation is a subjective method of examination, and these results may be influenced by the experience of the treating physician.

In our group of 68 patients, nipple/skin ulcerations were rather rare. Ulcerations present at the nipple level with malignancy as a starting point must be differentiated from benign pathologies and Paget’s Disease. In our group of patients, the cause of ulcerations was invasive breast carcinoma, stage G3.

In our group, 4 patients presented with nipple discharge. Of these, only one patient had serous-hemorrhagic nipple discharge. In our cohort, all patients who presented with nipple discharge were diagnosed with invasive breast carcinoma, grade G3. We can conclude that nipple discharge is a sign of gravity. In the clinical picture of breast cancer, nipple discharge accounts for 5% of all breast cancer symptoms and may be the earliest sign of breast cancer in some cases. In numerous studies, the incidence of nipple discharge in patients diagnosed with breast cancer is reported to be between 7 and 15% [45]. The most common cause of isolated nipple discharge of malignant origin is ductal carcinoma in situ. Another likely but much rarer cause is Paget’s Disease of the breast, a rare form of breast cancer that mainly affects the areola-breast complex.

Of the patients included in our study, 58 had positive axillary nodes. In all 58 positive cases, axillary nodes were present ipsilaterally. The status of locoregional lymph nodes is extremely important in determining the prognosis and treatment of patients with breast cancer. The presence of axillary lymph nodes is a predictor of increased risk of local and distant recurrence of breast cancer, directly affecting mortality. Thus, studies demonstrate that the overall survival rate is up to 40% lower for patients with positive lymph nodes compared to those with negative nodes [46]. The number of involved lymph nodes is traditionally used for post-surgical staging of breast cancer.

The American Joint Committee on Cancer classifies the staging of breast cancer anatomically and prognostically. Anatomical staging is based on the extent of the cancer and is translated into TNM staging: tumor size (T), lymph node status (N), and the presence of distant metastases (M). In our group of patients, the most frequently encountered anatomical TNM stagings are T2 N2 in 14.7% of the cases, respectively, and T2N3a in 13.23% of the cases.

Prognostic staging includes anatomical TNM staging, tumor grading, and tumor receptor status: HER-2, estrogen receptor, and progesterone receptor. Prognostic staging is preferably used in the context of patient care, being used in the United States of America for reporting all types of cancer patients. Furthermore, prognostic staging is divided into clinical and pathological groups. Pathologic staging applies to patients who have undergone surgery as the first treatment for breast cancer. This includes information used for clinical staging but also information obtained postoperatively from surgical resections. Pathological prognosis does not apply to patients who received neoadjuvant therapy. Thus, in our group of patients, the highest incidence of patients was in Stages IIIA, IIIB, and IV. We identified the lowest incidence for stage IIA. In numerous studies, the most frequently reported incidence was for stage IIIB, in 44.2%. For stage IIIA, the incidence was 4.5%. For IIIC, it was 12.6%, and for stage IV, it was 19.8%. The percentage of patients in stage IA was 5.4%. In stage IIA, it was 3.6%, and in stage IIB, it was 1.8%. It can be seen that the percentage of patients diagnosed in each stage is comparable to that in the specialized literature. In our patient group, there is a higher proportion of patients in stage IIIA (19% vs. 4.5%) and in stage IB (10% vs. 1.8%) [47,48].

The types of surgical interventions used for the 68 patients enrolled in this study were represented by Madden-modified radical mastectomy with radical axillary lymph node dissection, sectorectomy, subcutaneous mastectomy, and nipple-sparing mastectomy. We aim to extend the area of interventions with the help of our colleagues in the Plastic Surgery Unit so that we can perform mastectomy and breast reconstruction in selected patients and also by introducing the ICG combined with a radiotracer technique to perform sentinel lymph node excision.

Postoperative complications occurred only in patients in whom we performed the Madden technique with total excision of the axillary lymph nodes, and these complications were represented by lymphedema of the ipsilateral arm (6 patients), respectively, 3 cases that developed postoperative prepectoral seroma, which was conservatively treated. In the literature, the incidence of functionally significant lymphedema is nearly 20% after modified radical mastectomy. We can conclude that the incidence rate of postoperative complications during hospitalization was lower than that reported in the literature.

Regarding tumor histological grading, most patients were classified in Grade G2, i.e., intermediate histological grade, moderately differentiated, with a moderately favorable prognosis. A percentage of 44.41% of patients were classified in Grade G3, i.e., High Histological Grade, poorly differentiated with unfavorable prognosis. In numerous studies that also analyzed tumor grading, the reported incidence of Grade G1 tumors was approximately 23%, for G2 approximately 42%, and for G3 approximately 33%. Thus, we can conclude that in our group of patients there is a higher proportion of more advanced tumor grades, respectively, G2 and G3, unlike the specialized literature [47,48].

In our study group, the presence of HER-2 receptor positivity represented the majority. For our group of patients, there was no patient in whom the HER-2 receptor could not be determined, with the status remaining unknown. In the literature, the incidence of HER-2 positivity was 25.2%, the percentage of HER-2 negativity was 68.5%, and for 6.3%, the HER-2 status remained unknown because it could not be determined [49]. We can thus conclude that in our group of patients, the percentage of HER-2 positivity was higher than in the literature.

The estrogen receptor was positive in most cases. Numerous studies indicate the presence of ER in 71.2%. In 27.9%, estrogen receptors were negative, and in 0.9%, ER status remained unknown.

The most common histological tumor type encountered in our cohort was invasive ductal carcinoma (80.88%). This percentage is consistent with data from studies that also estimate the incidence of invasive ductal carcinoma at approximately 80%, being the most common type of breast cancer diagnosed. In our group, we encountered invasive lobular carcinoma in 10 patients (14.7%). In the literature, the incidence of this type of carcinoma is estimated at approximately 12% of patients diagnosed with breast cancer.

All our patients received postoperative chemotherapy, initiated approximately 3–4 weeks after surgery. In 9 patients, neoadjuvant chemotherapy was also considered. A well-executed selection of patients in whom neoadjuvant oncological treatment can be used gives the surgical intervention a higher success rate and, thus, a more favorable outcome for the patients.

Data from the literature indicate that Stage 0 and Stage I have a 100% 5-year survival rate. For cancers diagnosed in stages II and III, respectively, the 5-year survival rate is 93% and 72%. With the spread of the disease to the systemic level, the prognosis worsens dramatically. Among stage IV patients, only 22% will survive 5 years [50].

Currently, neoadjuvant chemotherapy represents the standard of care for patients with locally advanced breast cancer, being routinely used among patients with tumors smaller than 5 cm. Initially, chemotherapy was introduced to downsize locally advanced breast cancer and allow inoperable cases to be addressed surgically per primam. This has changed with the inclusion of initially operable patients in whom neoadjuvant chemotherapy is preferred for advancing disease stage, making tumor reduction possible and allowing for less extensive breast surgery in the future [51].

Breast cancer is a heterogeneous disease in which treatment must be well individualized, with several surgical solutions frequently available with the same oncological safety for certain patients. In these situations, it is important to include the patient in the therapeutic decision board through informed consent. For this to be optimal, the surgeon must be constantly updated with the available surgical options. The modern approach to surgical treatment continues constantly to become increasingly personalized for each patient and it must always depend on a multidisciplinary approach [52].

The diagnostic and treatment methods are constantly developing and improving, and let us take into consideration the role of AI (Artificial Intelligence), recently introduced in medicine, which, through its ability to analyze imaging investigations extremely quickly, but also through its ability to integrate a huge mass of information, it will certainly bring a plus in terms of the fight against cancer in general, and breast cancer in particular.

## 5. Conclusions

The prognosis of breast cancer diagnosed in the early stages is quite good, and the data in the literature show that over the past four decades, the survival rate for most patients diagnosed with breast cancer has improved, largely thanks to early detection and improved treatment.

The approach to surgical treatment varies depending on the particularities of each patient. However, the standard surgical procedure tends to continue to be represented by a modified radical mastectomy, which is also the most used surgical technique in the therapeutic approach to breast cancer in the General Surgery Clinic of the Colentina Hospital.

The current study has some limitations, represented by the small number of patients included that hinders analytical statistics comparing different treatments across patient groups or a significant comparison with literature data; also, the retrospective feature of the study prevents drawing conclusions, as some data may be lost compared to a prospective study. However, in the future, we intend to continue our study prospectively as well, with the aim of integrating our data into international multicenter studies and databases.

The main goal of the Romanian Ministry of Health is to provide, especially in rural areas, continuous information, as complete as possible, regarding, on the one hand, the severity of this pathology and, on the other hand, the imperative need for screening.

At the level of the Surgery Clinic of the Colentina Hospital, there is a permanent concern regarding alignment with international trends regarding the complex treatment of breast cancer, even if, sometimes, there are certain limitations regarding financial possibilities.

Based on the experience of our clinic, we can conclude that BCS is feasible for early-stage breast cancer (taking into account the tumor IHC, obtained either preoperatively or postoperatively), while, in advanced breast cancer, the best results were obtained after modified mastectomy with radical lymphadenectomy.

Our results align with those of the global studies, the only difference being that we were not able to perform sentinel lymph node biopsy instead of radical lymphadenectomy due to limited resources.

The three main future targets of the clinic are represented by:

- Acquiring the necessary technique for identifying the sentinel lymph node;
- Close collaboration with colleagues from the Plastic Surgery Department to perform, at the same surgical time, both the surgical time of breast tumor excision and the breast reconstruction since, through a program offered by the Ministry of Health, patients benefit from a breast prosthesis free of charge;
- Investment in AI technology, which, through its ability to quickly analyze imaging investigations and to integrate a huge mass of information, will certainly bring a plus in the fight against cancer in general and breast cancer in particular.

Regarding the evolution of surgical treatment and the future perspectives of treatment, we can conclude that to achieve the best therapeutic results in breast cancer, both multidisciplinary teams and permanent updates of knowledge in the field are necessary. With the help of these teams that include surgical oncologists, plastic surgeons, medical oncologists, radiation therapists, pathologists, imaging specialists, and very importantly, psychologists or even psychiatrists when needed, each patient’s case can be discussed individually, and a treatment plan can be formulated.

## Figures and Tables

**Table 1:** Location of the tumor.

**Upper-outer quadrant**	21 patients (30.88%)
**Upper-inner quadrant**	11 patients (16.17%)
**Lower-outer quadrant**	11 patients (16,17%)
**Lower-inner quadrant**	9 patients (13.23%)
**Central**	9 patients (13.23%)
**Multicentric localization**	7 patients (10.29%)

**Table 2:** Distribution of patients according to tumor size.

Size of the Tumor	Number of Patients
**1 cm**	12
**2 cm**	32
**3 cm**	16
**4 cm**	4
**5 cm**	3
**8 cm**	1

## Data Availability

No new data were created or analyzed in this study.
